# Benefits of global partnerships to facilitate access to medicines in developing countries: a multi-country analysis of patients and patient outcomes in GIPAP

**DOI:** 10.1186/1744-8603-5-19

**Published:** 2009-12-31

**Authors:** Panos Kanavos, Sotiris Vandoros, Pat Garcia-Gonzalez

**Affiliations:** 1 LSE Health, The London School of Economics and Political Science, London, UK; 2 The Max Foundation, Seattle, USA

## Abstract

**Background:**

Access to medicines in developing countries continues to be a significant problem due to lack of insurance and lack of affordability. Chronic Myeloid Leukemia (CML), a rare disease, can be treated effectively, but the pharmaceutical treatment available (imatinib) is costly and unaffordable by most patients. GIPAP, is a programme set up between a manufacturer and an NGO to provide free treatment to eligible CML patients in 80 countries worldwide.

**Objectives:**

To discuss the socio-economic and demographic characteristics of patients participating in GIPAP; to research the impact GIPAP is having on health outcomes (survival) of assistance-eligible CML patients; and to discuss the determinants of such outcomes and whether there are any variations according to socio-economic, demographic, or geographical criteria.

**Methods:**

Data for 13,568 patients across 15 countries, available quarterly, were analysed over the 2005-2007 period. Ordered Probit panel data analysis was used to analyze the determinants of a patient's progress in terms of participation in the programme. Four waves of patients entering quarterly in 2005 were used to evaluate patient survival over the sample period.

**Results:**

All patients in the sample are eligible to receive treatment provided they report to a facility quarterly. 62.3% of patients were male and 37.7% female. The majority (84.4%) entered during the chronic phase of the disease and their average age was 38.4 years. Having controlled for age, location and occupation, the analysis showed that patients were significantly much more likely to move towards a better health state after receiving treatment irrespective of their disease stage at the point of entry to the program (OR = 30.5, α = 1%); and that the larger the gap between diagnosis and approval for participation in the program, the more likely it is that patients' condition deteriorates (OR = 0.995, α = 1%), due to absence of treatment. Regressions to account for the effect of large countries (India, China, Pakistan) did not show any important differences when compared to the remaining countries in the sample. Survival analysis shows that at least 66 percent of all patients that entered the program in 2005 were alive and active by the end of 2007.

**Conclusions:**

GIPAP has a significant positive effect on patient access to important medicines for a life threatening condition such as CML. It impacts both the progress and phase of the disease and leads to a high survival rate. Overall, it sets a good example for access to treatment in developing countries, where such programmes can substitute or complement local efforts to provide care to eligible patients.

## Background and objectives

Patients suffering from life-threatening conditions in developing countries are often unable to access medicines that are critical for their treatment and survival. The high cost of medicines in relation to disposable income, low overall income and lack of health insurance coverage are determining factors of poor access, together with infrequent availability and poor quality [1]. It is estimated that in a number of transition countries and most developing nations, more than 80 per cent of pharmaceuticals are purchased out-of-pocket through both formal and informal means [2,3]. Beyond access to medicines, there are significant barriers to accessing services, including lack of available infrastructure, lack of diagnostic capabilities, and poor transport options, among others.

Chronic Myeloid Leukemia (CML) is a rare, life threatening condition affecting between one and two people per 100,000 annually. CML represents 15-20% of all cases of adult leukemia in Western societies. Frontline treatment of CML involves the use of Imatinib (Glivec^®^) (Appendix 1, Note 1). The beta crystal form of Imatinib has revolutionized the treatment and continued management of CML through precise molecular targeting; it appears to be more effective than Interferon-alpha (IFN-α) in terms of cytogenetic response (CR) and progression-free survival (PFS), with fewer side effects for patients in the chronic phase [4] and is also cost-effective compared to alternative therapy [5]. Studies have shown that, over a period of 5 years, a patient in the accelerated phase of CML will, on average, accrue an additional 2.09 Quality Adjusted Life Years (QALYs) with imatinib compared with conventional therapy, while patients in the blast-crisis phase will accrue an additional 0.58 QALYs compared with conventional therapy [6].

The Glivec International Patient Assistance Program (GIPAP) is a program set up by a manufacturer (Novartis) in partnership with an NGO (The Max Foundation - TMF) in collaboration with other NGOs such as the China Charity Foundation and Axios International, to facilitate access to and distribution of imatinib directly to patients through their providers. GIPAP aims to fill the gaps of imperfect access to eligible patients in developing countries that cannot afford this costly treatment [7]. Under the program, the manufacturer provides the drug at no cost directly to eligible patients identified and selected by TMF in participating countries. This is not the only global partnership that helps provide important medicines to people who cannot afford them in developing countries [8,9]. The International Trachoma Initiative (ITI) helps the implementation of plans to eliminate blinding trachoma [10]. The Mectizan partnership, involving a pharmaceutical manufacturer (MSD), the World Bank, governments and NGOs was set up in order to provide ivermectin to patients in developing countries [11]. The Accelerated Access Initiative (AAI) involves seven research-based pharmaceutical manufacturers and five United Nations partners aiming to provide better access to anti-retroviral (ARV) drugs in developing countries; by the end of December 2005, more than 716,000 people living with HIV/AIDS in developing countries were receiving treatment with at least one ARV medicine provided by the AAI [12]. The World Health Organization has set up guidelines which must be followed in these cases. GIPAP also complies with these guidelines.

Currently, 80 countries worldwide take part in GIPAP and the total number of active CML patients benefiting from this initiative reaches 18,000 worldwide. Table 1 shows the breakdown of participating regions and the CML active patients per region.

**Table 1:** Geographical distribution of GIPAP active^1 ^participating patients, 2007

Continent	Population^2^	Number of active CML patients	% of population active
Asia	3,461,233,811	14,927	0.0004%
Africa	643,500,700	1,201	0.0002%
Latin America	371,975,205	1,370	0.0004%
Europe	245,085,256	509	0.0002%
Oceania	839,000	7	0.0008%

Total	4,722,633,972	18,004	

***Note: ***^1 ^If the number of non-active patients is added, the total number of patients participating in the program reaches 26,532.

^2 ^Population figures are related to participating countries only.

***Source: ***The authors from the GIPAP database.

In many participating countries, particularly those in sub-Saharan Africa, GIPAP is the only source of available treatment for CML, as there is no state health insurance and very few people can afford private health insurance or the out-of-pocket expense to acquire the needed medication. As a result, GIPAP may often cover all patients diagnosed with the condition irrespective of the type of facility they are diagnosed in (Appendix 1, Note 2). There are no restrictions in the number of patients eligible for GIPAP in any participating country and, as this is a program requiring significant physician input, new patients are enrolled as long as a qualified physician assumes responsibility for them. In countries such as Argentina and Chile, the program is supplementary to state insurance coverage, the latter being limited only to certain population groups (e.g. government officials).

In order to administer imatinib to eligible patients, the manufacturer identifies the appropriate medical centres and physicians and supplies the drug to these centers. These centres have been certified to comply with minimum services to CML patients. Reports for each patient are filled in quarterly by physicians and sent to TMF's headquarters. A requirement for a physician to participate in the programme is that s/he has an internet connection. TMF conducts socio-economic evaluations of patients, guides physicians through the patient evaluation process, and provides emotional support, information and referral assistance to patients, their families and care givers. It also monitors patients to support the highest standard of patient care, helps identify and qualify eligible medical centers and physicians worldwide, and protects confidential patient information and data received during the implementation of the program. A program with such broad geographical expanse cannot work exactly in the same way in all regions or countries. Thus, the operational requirements of GIPAP vary from country to country or region to region.

While GIPAP has been in operation for several years, thus far, assessment of the impact it is having on CML patients in developing countries has at best been anecdotal or relied on individual physician opinions [13]. In this paper we aim to, first, identify and discuss the socio-economic and demographic characteristics of the patients participating in GIPAP, and, second, to analyze the impact the program is having on health outcomes of CML-diagnosed patients, discuss the determinants of such outcomes and whether there are any variations according to socio-economic, demographic, or geographical criteria. In doing so, the paper also discusses the policy implications regarding access to medicines in developing countries. Section 2 discusses the methodology employed in the paper; section 3 presents and section 4 discusses the results; finally, section 5 draws the main conclusions.

## Data and Methods

### Data

In order to address the above objectives, we focused on CML using data collected by TMF as part of its global remit to implement GIPAP. The manufacturer establishes socio-economic criteria modeled on the World Health Organization guidelines for charitable donation programs as well as medical criteria determining patient eligibility for GIPAP, while TMF reviews patient applications for participation in the program and collects data and information on each patient in the program based on physician records and assessment. As a result, TMF has exclusive responsibility and oversight in setting up, running and monitoring the program (Appendix 1, Note 3).

Patient eligibility is determined primarily on the basis of diagnosis as well as income/socioeconomic status, as follows: (a) GIPAP helps patients who are properly diagnosed with Philadelphia chromosome-positive chronic myeloid leukemia (Ph+ CML) and patients with c-Kit (CD117)-positive inoperable and/or metastatic malignant gastrointestinal stromal tumors (GISTs) (Appendix 1, Note 4); and (b) GIPAP provides assistance to patients who are not insured or reimbursed, cannot pay for treatment privately, and live in countries that have minimal reimbursement capabilities for their condition. Based on these criteria, it is possible that GIPAP covers all those diagnosed with CML in certain countries because of their low income level.

Data were extracted from the TMF database covering the period from the beginning of 2005 to the end of 2007 on a quarterly basis ensuring that all patients had first entered in the first quarter of 2005 or later. Thus, the study period comprised 12 quarters. Patients that entered the program before this date were excluded as the objective was to study patients from the moment of their entry in the program. The study included 15 countries in the analysis, namely, Kenya, Nigeria, South Africa and Sudan from Africa; Argentina, Chile, El Salvador and Mexico from Latin America; Russia and Georgia from Europe; China, India, Malaysia, Pakistan and Thailand from Asia. In the Chinese context, each of China's provinces and municipal entities has its own healthcare infrastructure. This necessitates varying reimbursement schemes for Imatinib, including shared contribution and co-pay models.

Country selection was based on geography, ensuring representation from all continents where the program operates, the size of eligible population, program penetration (percentage of participants in total patient population) and health insurance program availability for some segments of the population. This resulted in the total number of patients being 13,568 across the selected countries and for the study period (Appendix 1, Note 5). Of these patients there was no information about the initial phase for only 3 patients and no information about the latest phase for 20 patients. When taking into account the time dimension, the total sample size was N = 66,681 observations during the study period 2005-2007. The sample includes the largest GIPAP participant (India), other large Asian countries (Pakistan, Thailand, Malaysia and China), countries with some health insurance coverage for small segments of the population, (Russia, Argentina, Chile, Mexico and Georgia), African countries with large populations (South Africa, Nigeria, Kenya and Sudan) and representative Latin American countries such as Mexico, Argentina and El Salvador.

TMF collects feedback from the participating physicians electronically and on a regular basis; in addition patients must be medically re-approved on average four times per year in order to continue receiving treatment. As a result, the frequency of data is quarterly. If a patient does not present himself for their quarterly review, they are considered as "closed" or non-active, but they can be re-instated in the program if contact with them is re-established. Aside from monitoring eligible patients closely, this also enables longitudinal analysis.

The patient-related data are sent from local GIPAP participating physicians to the central database quarterly. The data are stored in the central system at TMF headquarters in Seattle. All data are anonymous and are uniquely identified by a code number. The data for this study were accessed through the IT services at TMF, by country, patient and other demographic characteristics. The unit of analysis is always the patient.

### Dependent Variables

The variable used as a proxy of patient performance within GIPAP is a patient's current phase (*curphase*). Current phase refers to one of the different phases a patient may be in: Blast Crisis, Accelerated, Chronic or Remission. A number was assigned to each phase: 1 for blast crisis, 2 for accelerated phase, 3 for chronic phase and 4 for remission, making this a discrete variable. In this categorization the lowest number represents the worst possible clinical state (blast crisis) and the highest number denotes the best possible clinical state (remission).

### Explanatory Variables

A number of explanatory variables were included in the analysis, as follows: *Origphase *refers to the patient's health or clinical state upon their admission to the program; it can therefore be categorized as 1 for blast crisis, 2 for accelerated phase and 3 for chronic phase. The study period commenced in quarter 1, 2005, and all patients active before that date are excluded from the analysis.

*Age at approval *is the age of the patient at the time of their admission to the program. *Quarter *(1-12) refers to time, starting with the first quarter of 2005 till the fourth quarter of 2007, a total of 12 quarters. This is a control variable used to capture unobserved heterogeneity, factors which change over time and cannot be included in the vector of explanatory variables and enables to control for natural changes in patient outcomes over time. There are many factors that tend to change over time and the inclusion of a time variable eliminates this effect. *Gender *is a dummy variable, which takes the value of 0 for men and 1 for women. *Gap *denotes the number of months from the confirmation of the diagnosis date to the approval of participation in GIPAP.

*Close *indicates whether a patient has been closed at least once over the period he has been participating in the program. It is a binary variable taking the value of 0 and 1, 0 indicating that the patient has never been closed, 1 indicating that patient has been closed at least once. A patient is considered to be closed when s/he has not reported for treatment to his/her designated centre for 1 quarter. Consequently, this does not refer to the present status of the patient as active or closed. It is used as a control variable to capture unobserved characteristics of a patient who has not always been present in the program. Patients may be classified as "closed" for a variety of reasons, including (a) inability to keep track of the patient, (b) the patient not making the journey to the clinic where treatment takes place, (c) the patient not showing up on the specified day of their treatment, (d) inability to contact the patient, (e) becoming ineligible to receive treatment through GIPAP and (f) death. It is also possible that individual patients may be "closed" more than once and re-appear in the database, as patient participation depends on receipt of treatment and monitoring on a quarterly basis. With the exception of death, none of these reasons prevent patients from re-entering the program in the next period once they show up for their treatment. Thus, closures are a source of potential bias in the data but they by no means imply that the patient is deceased. Whereas patients who were closed but re-entered the program did not die, for those who do not re-appear in the dataset till the end of the study period we cannot be certain about their status. This is a limitation of the available data. Overall, there does not appear to be any seasonal effect in the number of closed cases.

*Ins *is a dummy variable, taking into account whether a country offers universal health insurance coverage to part of its population. Four countries (The Russian Federation, Argentina, Chile and Georgia) offer universal health insurance coverage - although by no means comprehensive - to their population, whereas all other study countries do not. Although this does not apply to the GIPAP-eligible population, as the latter is selected based on inability to pay, this variable is used as a control to explain any heterogeneity in the data. For instance, it could be argued that the presence of universal health insurance indicates better features of the health system as a whole. Health planning and improved geographical access could be part of a program which includes health insurance for a significant part of the population.

In addition, 14 country dummies are included, one for each country, in order to control for any country-specific effects as well as evaluate how the program compares across countries. Finally, 13 dummy variables have been introduced to identify patient occupation: *Undefined*, *agriculture*, *business*, *education*, *government*, *health and social work*, *manufacturing*, *other*, *retired*, *self employed*, *student*, *transport *and *unemployed*. Profession also captures other, unobserved patient characteristics, such as education, medical sophistication and lifestyle. Table 2 presents all variables included in the analysis and their definition.

**Table 2:** Variables and definitions

Variable		Mean	Std. Dev.
*Quarter*	Quarter (time). Indicates the number of quarters a patient has been participating in GIPAP	8.347	2.939

*Origphase*	Original Phase of Patient: 1 for Blast Crisis, 2 for Accelerated, 3 for Chronic, 4 for Remission	2.821	0.488

*Curphase*	Current Phase of the Patient: 1 for Blast Crisis, 2 for Accelerated, 3 for Chronic, 4 for Remission	2.924	0.554

*Ins*	Dummy variable. Indicates whether there is universal health insurance coverage or not; 1 for Argentina, Chile, Georgia and Russia, 0 for the other countries.	0.039	0.193

*Ageatapproval*	Age of the patient at his or her approval for participation in GIPAP	38.443	14.147

*Gender*	Gender. Dummy variable; 0 for male, 1 for female	0.372	0.483

*Gap*	Time gap between Diagnosis and Approval. It is the difference in months between the date of diagnosis of the patient suffering from CML and the date of approval for participation in GIPAP	4.617	8.997

*Close*	Dummy variable. Indicates whether a patient is considered closed or not. 0 for not closed, 1 for closed.	0.056	0.231

*Argentina*		0.007	0.083
*Chile*		0.012	0.109
*China*		0.160	0.367
*El Salvador*		0.004	0.067
*Georgia*		0.008	0.086
*India*		0.520	0.500
*Kenya*		0.008	0.089
*Malaysia*		0.038	0.191
*Mexico*		0.028	0.165
*Nigeria*		0.009	0.092
*Pakistan*		0.103	0.303
*Russia*		0.012	0.110
*South Africa*		0.022	0.145
*Sudan*		0.026	0.158
*Thailand*		0.045	0.207
*Undefined*		0.068	0.252
*Agriculture*		0.229	0.420
*Business*		0.044	0.206
*Education*		0.019	0.136
*Government*		0.035	0.183
*Health-Social*		0.011	0.104
*Hospitality*		0.006	0.077
*Manufacturing*		0.040	0.195
*Other*		0.291	0.454
*Retired*		0.039	0.193
*Self Employed*		0.085	0.279
*Student*		0.029	0.168
*Transport*		0.013	0.115
*Unemployed*		0.091	0.288

***Source: ***Authors' compilations from GIPAP database.

### Model Specification

Based on the discussion in the previous section, the model that is considered for empirical analysis has the following specification:(1)

Equation (1) has current phase as the dependent variable. The values assigned to the current phase increase as the outlook for the condition improves.

### Estimation Method

In order to assess the effect GIPAP is having on CML patients, both descriptive and econometric analysis are pursued.

Descriptive statistics show the distribution of patients, their phase from the moment they are accepted in the program and an understanding of demographics. These also show how characteristics change after entering the program.

Panel data are used to conduct the econometric analysis. The use of panel data is justified because it controls for unobserved heterogeneity across individuals. The nature of the patient-specific data allows the reasonable assumption to be made that unobserved heterogeneity is uncorrelated with the included variables.

In this analysis, the special (ordinal) nature of the dependent variable dictates the use of the ordered model. A Panel Data Ordered Probit (OP) model is therefore used in this case, as the dependent variable is not continuous. Discrete values are assigned to different groups of observations, which change as patients' reported health state changes. The OP model is preferable to the Ordinary Least Squares (OLS) model because in the latter the variance of the error term is not constant and is dependent upon the explanatory variables [14]. The dependent variable is thus ordered in such a way that imposes the use of the OP model: An OP model treats differences between discrete outcomes as constant [15]. In this model, the dependent variable has a logical ordering. Current phase is ordered in a logical sequence, depending on the severity of the disease, assigning different values to the different phases: 1 for blast crisis, 2 for accelerated phase, 3 for chronic phase and 4 for remission, as identified in the literature. The order assigns an increasing number for a better condition. The odds ratio (OR) shows the probability of the patient moving to a higher phase, over the probability of the patient moving to a lower phase. In other words, the odds ratio shows, for a unit change in the regressor, the odds of a higher phase compared to a lower phase are changed by a factor of the independent variable, other things being equal.

### Survival analysis

The study period and the longitudinal nature of the data enables the assessment of the number of patients that remained active up to 3 years after they first entered the program (2005 - 2007). The definition of "active patients" means that these patients continue to be registered in the program and benefit from the treatment provided. Consequently, this enables the measurement of survival at individual patient level. In order to examine this, 4 waves of new patients were isolated and studied, each wave entering quarterly in 2005. By following these patients through to the end of the study period, it was possible to calculate how many would benefit from GIPAP and observe the attrition rate over three years. A survival rate was calculated as the ratio of those continuing to receive medication over the total number of patients that entered originally.

## Results

### Descriptive Statistics

A summary of the descriptive statistics from the 15 study countries is shown in Table 3. Of all participating patients, 62.3% are male. This is consistent with findings in other settings that CML is a disease affecting men more frequently than women. According to the National Cancer Institute, CML affects 1.9 per 100,000 men and 1.1 per 100,000 women in the United States. The average age at diagnosis in this study is 38.7 years, which is significantly lower than similar patient cohorts in developed countries. In the United States the average age at diagnosis over the 2001-2005 period was 66 years [16]. The dominant age group in the study is age band 31-40 years (26.9%), followed by 41-50 years (21.5%) and 21-30 years (20.5%). At the time of initial diagnosis, 11,414 patients (84.14%) were in the chronic phase, 1,229 (9.05%) were at the accelerated phase and 923 (6.8%) were in blast crisis. No patients enter GIPAP in the "remission" phase; patients in remission reach that stage after receiving treatment.

**Table 3:** Summary Statistics at Patient Level, 2005 - 2007

Participants (Total)			13,568	
Average Age (years)			38.69	
Average Time Gap between Diagnosis and Approval (months)			4.61	
				
**Age Group**	**No. of patients**		**No. of observations**	
0-20	1,332	9.82%	6474	9.71%
21-30	2,786	20.53%	13,970	20.95%
31-40	3,646	26.87%	18,273	27.40%
41-50	2,914	21.48%	14,191	21.28%
51-60	1,895	13.97%	9,185	13.77%
61-70	758	5.59%	3594	5.39%
71+	237	1.75%	994	1.49%
Total	13,568	100.00%	66,681	100.00%
				
**Gender**				

	**No. of patients**		**No. of observations**	
Male	8,453	62.30%	41,873	62.80%
Female	5,115	37.70%	24,808	37.20%
Total	13,568	100.00%	66,681	100.00%
				
**Original Phase**				

	**No. of patients**		**No. of observations**	
Chronic	11,414	84.14%	57,728	86.57%
Accelerated	1,228	9.05%	5,958	8.94%
Blast Crisis	923	6.80%	2,995	4.49%
Total	13565	100.00%	66,681	100.00%
				
**Current Phase**				

	**No. of patients^1^**		**No. of observations**	
Chronic	N/A		54,192	81.27%
Accelerated			4,587	6.88%
Blast Crisis			2,794	4.19%
Remission			5,108	7.66%
Total			66,681	100.00%
				
**Status**				

	**No. of patients^1^**		**No. of observations**	
Closed	N/A		3,734	5.60%
Active			62,947	94.40%
Total			66,681	100.00%

***Note: ***^1 ^Although we do have data at patient level, we cannot report summary statistics here, as this changes per patient over time. Thus, statistics are only reported per observation, not per patient.

***Source: ***Authors' compilations from GIPAP database.

### Results of the Econometric Analysis

In order to account for the factors that determine the progress of a patient diagnosed with CML, an Ordered Probit panel data econometric model was estimated. The panel identifier is the individual patient, as there exist multiple observations for each patient at different points in time. Following the model in equation (1), the dependent variable was the current health state of the patient such that the higher the number assigned to the current state, the better the patient's overall condition or outlook. The explanatory variables included different socio-economic factors and demographics.

The original phase is positively and significantly associated with the current phase (OR = 30.5, α = 1%), indicating that patients classified as chronic in the original phase were significantly much more likely to improve over time (more likely to move towards a better health state); comparable effects could be seen for those patients in the accelerated phase or blast crisis in the original phase. This is consistent with expectations and the results shown in the previous section, as patients whose initial condition was better are expected, on average, to be doing better at later stages, once they enroll on the program. This also suggests that, overall, the program contributes to patient health improvement.

Patient age at approval is not statistically significant and the OR is very close to 1. Results for gender show that this variable is not statistically significant either. The time gap between diagnosis and approval for entry into the program is statistically significant. The larger the gap between diagnosis and approval for GIPAP participation, the more likely it is that the patient's condition deteriorates (OR = 0.995, α = 1%). This is explained by the fact that during this time gap most patients would not have access to treatment. As a result, shortening this gap over time may have contributed to fast access to medical treatment by eligible patients.

The insurance variable does not yield statistically significant results, which is expected, as GIPAP participation is contingent on patients not having insurance coverage. Insurance was used as a control variable to take into account any unexplained heterogeneity between countries that offer health insurance coverage and countries that do not, as well as features that a health system with health insurance is expected to have. Such features may be better health planning and improved geographic access.

The country dummies help control for the country effect and any other regional differences that are not captured by other variables. Only Argentina, Georgia, Kenya, Malaysia and Sudan differ from the reference country (Thailand). This shows how patients are more likely to do in terms of progress with their disease in different countries. The effect of occupation is a set of 13 dummy variables showing that skilled workers (business, education, government) and the self-employed are significantly more likely to move to a better health state than less skilled workers or retirees.

The model results presented in Table 4 relate to all 15 countries; three of these countries account for 78.3% of the total number of observations (India, 52% of the total sample; China, 16%; and Pakistan, 10.3%). As a small number of countries dominate the sample, there may be potential for bias in the results. In order to account for this, separate estimations have been produced for India, China, Pakistan, as well as for the remaining 12 countries together in order to determine if there are significant differences in the results. These are presented in Table 5. The original phase is statistically significant in all four cases, and towards the same direction, showing that enrolment on the program can improve patient outlook (OR>1, α = 1%). Age, gender, the time gap between diagnosis and approval and whether a patient is closed or not also have the same effect across all 4 separate regressions. Overall, the results across the four different models appear to be in the same direction, and consistent with those in the aggregate sample, suggesting that the factors influencing patient progress are similar across the countries in the sample, and that the inclusion of a large number of observations from just 3 countries does not create a bias.

**Table 4:** GIPAP: Results of a Random Effects Ordered Probit Model

Dependent Variable	***Curphase***	
	Odds Ratio	SE
*Quarter*	1.071***	0.004
*Origphase*	30.508***	0.036
*Ins*	0.989	0.062
*Ageatapproval*	0.999	0.001
*Gender*	1.050	0.036
*Gap*	0.995***	0.002
*Close*	0.652***	0.039

*Argentina*	3.561***	0.206
*Chile*	1.018	0.201
*China*	1.081	0.098
*El Salvador*	0.819	0.220
*Georgia*	2.773***	0.260
*India*	1.182	0.098
*Kenya*	0.285***	0.263
*Malaysia*	1.605**	0.187
*Mexico*	0.880	0.120
*Nigeria*	0.619*	0.263
*Pakistan*	0.839	0.109
*Russia*	0.832	0.154
*South Africa*	1.010	0.144
*Sudan*	13.237***	0.202

*Undefined*	2.179***	0.153
*Business*	1.394***	0.086
*Education*	1.330*	0.124
*Government*	1.259*	0.100
*Health-Social*	0.931	0.130
*Hospitality*	1.266	0.177
*Manufacturing*	1.081	0.079
*Other*	0.984	0.050
*Retired*	1.085	0.100
*Self Employed*	1.247***	0.068
*Student*	1.181*	0.094
*Transport*	1.269	0.221
*Unemployed*	1.112	0.065

Log Likelihood	-19792.852	
LR chi2(31)	9836.92	
Observations	66,681	

Note: Significance levels: *** indicates significance at 1% level; ** at 5% level and * at 10% level

**Table 5:** GIPAP: Results of a Random Effects Ordered Probit Model - country breakdown

	Country Regressions							
Model	India		China		Pakistan		Remaining 12 countries	
Dependent Variable	*Curphase*		*Curphase*		*Curphase*		*Curphase*	
	Odds Ratio	SE	Odds Ratio	SE	Odds Ratio	SE	Odds Ratio	SE
*Quarter*	1.069***	0.006	1.089***	0.009	0.983	0.015	1.093***	0.006
*Origphase*	240.567***	0.104	21.052***	0.063	115.816***	0.165	18.102***	0.073
*Ageatapproval*	1.005***	0.002	0.998	0.002	1.016***	0.004	0.994***	0.003
*Gender*	1.012	0.057	1.021	0.066	1.141	0.106	0.933	0.063
*Gap*	0.996	0.004	1.001	0.003	0.985**	0.007	0.990***	0.003
*Close*	0.685***	0.086	0.691***	0.088	0.404***	0.196	0.694***	0.059

Log Likelihood	-6663.125		-4495.819		-1277.437		-6638.452	
LR chi2(31)	4401.33		2278.68		660.41		1612.61	
Observations	34,686		10,663		6,837		14,493	

Note: Significance levels: *** indicates significance at 1% level; ** at 5% level and * at 10% level.

### Survival over time

**Figure 1:**
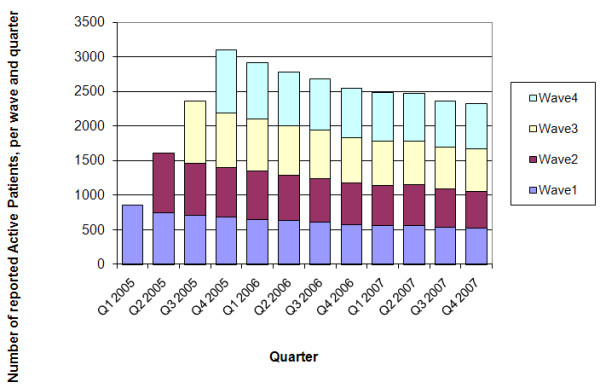
**Active Patients, Waves 1, 2, 3, 4**. ***Source: ***The authors from GIPAP.

In total, 3,529 active patients entered GIPAP in the four quarters of 2005. Figure 1 shows their progress and gradual attrition quarterly over the 2005 - 2007 study period.

Table 6 summarises the number of active patients per wave and shows the patient attrition on a quarterly basis as well as the number of active patients remaining at the end of each quarter. Examining the first wave of patients during the first quarter of 2005 (Q1 - 2005), a total of 850 active patients entered GIPAP. For these patients, there is a total of 7,596 observations over the 3-year period (12 quarters), corresponding to an average of 8.94 observations per patient. Given this natural ceiling, more than 2 years average active period per patient is very high (as many will carry on being active beyond the study period). This indicates that for many patients CML becomes a chronic condition and that those benefiting from imatinib are able to return to their activities and in principle continue to contribute to their families and the local economy. In Q4 2007 (after 3 years) 520 active patients remain from the first wave. Thus, out of 850 active patients who started in Q1 2005, 520 were still reported in the database and were active after 3 years, corresponding to 61.2% of the original patient total. Of the total number of patients who entered in each quarter of 2005, at the end of the study period (after 12, 11, 10 and 9 quarters for waves 1, 2, 3 and 4 respectively), in total 2,317 patients remained active in the fourth quarter of 2007, corresponding to a 66% survival rate.

**Table 6:** GIPAP: 3-year survival for patients entering the programme in 2005

	2005				2006				2007			
	**Q1**	**Q2**	**Q3**	**Q4**	**Q1**	**Q2**	**Q3**	**Q4**	**Q1**	**Q2**	**Q3**	**Q4**

Wave 1 (entering quarter 1, 2005)												

Active	850	742	701	680	649	626	603	576	559	557	533	520
Not reported/Closed		108	149	170	201	224	247	274	291	293	317	330
Total	850	850	850	850	850	850	850	850	850	850	850	850

Wave 2 (entering quarter 2, 2005)												

Active	0	866	752	717	695	655	630	594	574	588	557	537
Not reported/Closed			114	149	171	211	236	272	292	278	309	329
Total	0	866	866	866	866	866	866	866	866	866	866	866

Wave 3 (entering quarter 3, 2005)												

Active	0	0	910	795	752	723	704	664	649	639	608	606
Not reported/Closed				115	158	187	206	246	261	271	302	304
Total	0	0	910	910	910	910	910	910	910	910	910	910

Wave 4 (entering quarter 4, 2005)												

Active	0	0	0	903	821	775	745	710	695	680	656	654
Not reported/Closed					82	128	158	193	208	223	247	249
Total	0	0	0	903	903	903	903	903	903	903	903	903

**All 4 waves (quarters 1 - 4, 2005)**												

**Total Active**	**850**	**1608**	**2363**	**3095**	**2917**	**2779**	**2682**	**2544**	**2477**	**2464**	**2354**	**2317**

**Total Not reported/Closed**	**0**	**108**	**263**	**434**	**612**	**750**	**847**	**985**	**1052**	**1065**	**1175**	**1212**
**Total number of patients**	**850**	**1716**	**2626**	**3529**	**3529**	**3529**	**3529**	**3529**	**3529**	**3529**	**3529**	**3529**

***Source***: The authors from GIPAP.

**Figure 2:**
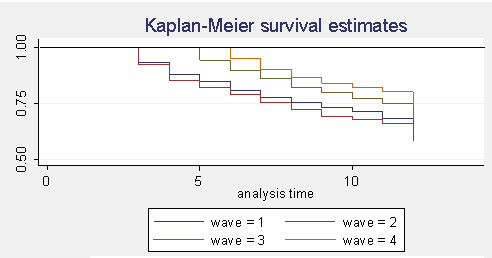
**Survival Analysis (2005 - 2007, quarterly)**. Kaplan Meier Survival Estimates.

Figure 2 demonstrates the Kaplan - Meier survival estimates. The Kaplan-Meier survival probability is the fraction of the number of patients surviving in each quarter over the number of patients at risk. The probability of surviving to any point is estimated from the cumulative probability of surviving each of the preceding time intervals. Thus the graph shows the fraction of the population which survives over time. The four waves are graphed separately on the same axes, showing how patients in each wave perform. When comparing survival across the four waves, the 9-quarter survival ranges between 65.8% (wave 1, covering the period from 2005, Q1, to 2007, Q1) and 72.4% (wave 4, covering the period from 2005, Q4, to 2007, Q4). By the end of the study period (2007, Q4), 66% of all CML patients remain active and are shown to be receiving treatment under GIPAP. This compares favourably with the IRIS clinical trial data, despite the difficulties in delivering care in developing countries. Further, many cases are lost track of, indicating that patients may survive, which would lead to even higher survival rates (Appendix 1, Note 6).

## Discussion & Policy Implications

Access to medicines in developing countries continues to be adversely affected by poverty and the lack of adequate statutory health insurance coverage to local populations. Additional predicaments include the poor state of health facilities as well as geographical disparities in their availability, which further hamper patient access. Patients diagnosed with CML are no exception to the above problems. Although treatments such as imatinib are in principle available on the private market, the out-of-pocket acquisition cost is prohibitive for most developing country patients, as the annual drug treatment cost may exceed $36,000 [17]. Even in cases where a generic version of branded imatinib becomes available, the out-of-pocket cost continues to be unaffordable for the vast majority of patients. Treatment alternatives to imatinib require specialized care, which may be expensive, not available within easy reach, and with uncertain outcome.

The findings of the study suggest that patients are significantly much more likely to move towards a better health state after receiving treatment irrespective of their disease stage at the point of entry to the program and that the larger the gap between diagnosis and approval for participation in the program, the more likely it is that patients' condition deteriorates due to absence of treatment. Under the auspices of GIPAP, CML patients are granted free medical treatment, reducing total health costs significantly, potentially helping patients return to normal activity and contributing to life extension. This becomes even more important when taking into account that the average age of CML patients in developing countries is significantly lower than that in developed countries [18], suggesting that GIPAP helps patients in very productive ages. Demonstrating benefit has obvious positive societal implications for patients and their families in terms of ability to work and contribution to family income. The 3-year survival was found to be at minimum 66% of the originally enrolled patients across the 15 study countries and this compares favorably with other studies in the developed world [18]. This is also a strong indication that the program provides a sustainable health benefit and that patients return for their treatment at regular intervals.

The success of GIPAP depends on whether and how patients' lives are extended by participation in the program. If at least two thirds of patients who originally registered in the program and suffering from this life-threatening condition are still participating after a 3 year period, this is a strong indication that the program delivers care and helps patients stay alive. This rate is likely to be an underestimate of true overall survival because of the likely biases in the attrition rate and the number of patients classified as closed. Because of the definition of "closure", the attrition rate includes patients who may not have died and may still be receiving the treatment through other sources. As a result, the survival rate obtained is the minimum survival rate of patients in the study period.

GIPAP seems to fulfill the critical role of enabling access to very poor patients and providing a life-saving treatment that extends life. Many GIPAP participants go into remission after receiving imatinib through the program, while they would otherwise be unable to access the treatment themselves and face deteriorating health. Participation in the program is voluntary, and some patients may choose to drop out; although this is not a desirable outcome, a patient cannot be prohibited from discontinuing the treatment, despite the impact this may have on further patient follow-up, adherence, overall cost and long-term survival [19].

Close monitoring of patients is critical in order to achieve the maximum possible adherence and maximize impact. Given that many patients live in isolated, remote areas, with limited access to their participating physician or hospital due to distance or unaffordable travel costs, the effectiveness of the program may be adversely affected compared with a situation where patients have easy access to health facilities and professionals. This is compatible with other comparable findings [20] and in order to alleviate what seems to be a health problem compounded by poverty, local governments can assist by improving infrastructure and communications, in order to maximize the benefit of programs such as GIPAP.

GIPAP coincides with the appearance and subsequent proliferation of Global Health Partnerships (GHPs) in the last decade or so, which have amassed significant support among bilateral and multilateral donor agencies [9] and have included product supply initiatives such as the ITI and the AAI. There are arguments favoring GHPs over bilateral or multilateral aid, which also apply in the case of GIPAP and include (a) flexibility in terms of organizing and delivering care where needed; (b) scale economies; (c) country links, enabling delivery of care and assistance in a timely fashion; (d) independence from country-specific structures as well as donor country preferences; and (e) their effectiveness in terms of raising and using aid [21]. As a drug donation scheme, GIPAP must fulfill and adhere to certain criteria set up by the World Health Organization on drug donations [22] and good pharmaceutical procurement [23].

Drug donation and distribution programs such as GIPAP may provide an alternative platform for drug access to eligible patients in developing countries. By building on existing structures in health facilities and mobilizing clinicians at no additional cost to local health systems, GIPAP enables access to a life-saving medication at no cost to patients or the local health care systems and transforming a life-threatening disease into a chronic condition. The distribution of the medicine from the manufacturer to the patient via the associated NGO provides a practical solution which also avoids other potential problems associated with drug donations and distribution channel shortcomings in both developing and transitional country contexts. Indeed, evidence suggests that although humanitarian donations have been an important source of medicines for many countries, the donated drugs may not reach their intended targets, namely the patients [24-26].

Overall, assuming there is continuity in the program over the long-term, that it is not an opportunistic venture and that the drug donation segment is compliant with the WHO guidelines for drug donations, programs such as GIPAP, resulting from a partnership between different stakeholders, could provide alternative models of enabling access, providing effective and targeted health care and making healthcare affordable to patients in developing countries in the absence of a formal health insurance coverage system.

The preceding analysis is not without limitations. The dependent variable is subjective and based on physician assessment, the latter being subject to time pressure; this may lead to limitations when examining the factors influencing patient current phase. Additionally, the data do not record with precision the reason for all "closed" cases. Although it may not be possible in all developing country settings to follow all patients, not knowing the precise reason for patient drop-outs can only provide an estimate of survival analysis. Finally, it may be the case that the results of the study may not be generalizable to the other countries that GIPAP covers.

## Conclusions

GIPAP has been offering free treatment to CML patients in developing countries since 2002. This is an example of patient assistance in countries where very little or no health insurance or prescription drug coverage exists and patients cannot afford appropriate treatment. Such initiatives can work as a complement to existing public health insurance schemes and help people fight life-threatening conditions such as CML.

We have empirically examined the determinants of patients' progress in GIPAP. The empirical model has helped observe the effects of various characteristics on the phase patients are in and helped detect the differences across participating countries. The survival analysis showed that the majority of GIPAP participants remain in the program for a long time, clearly underlining its effect on transforming a life-threatening disease into a chronic one. Access issues are not evident because GIPAP is in itself a program facilitating access.

Future research could include data from CML patients in developed countries, and compare the outcomes of treatment of insured patients in developed countries to GIPAP participants. This would show how the program works compared to countries with regular health insurance and would test the possibility of such a patient assistance program to act as a substitute for health insurance.

## Competing interests

This research was funded via an unrestricted educational grant from Novartis.

## Authors' contributions

Study conception and design: PK. Data requirements: PK, PGG, SV. Data acquisition and extraction: SV, PGG. Analysis and interpretation of data: PK, SV. Drafting of manuscript: PK, SV. Critical revision: PK, SV, PGG. All authors read and approved the final manuscript.

## Appendix 1

Note 1. Imatinib is also used for the treatment of metastatic malignant gastrointestinal stromal tumors (GIST).

Note 2. In all countries where GIPAP operates, centres are selected where patients can be offered treatment and be monitored. The criteria for selecting these centers include having diagnostic capabilities and having had previous experience treating CML. In many countries the GIPAP Qualified Institution is the only institution able to diagnose or with the experience to treat CML. This is the case particularly in many African countries where there is one cancer center in a single country. However, patients do not necessarily have to have been diagnosed in GIPAP Qualified Institutions to be offered treatment and enroll onto the program.

Note 3. TMF has an agreement with each of the GIPAP physicians and Institutions in all countries it operates. A Memorandum of Understanding (MOU) between TMF and each physician is signed upon approval of each physician as a GIPAP qualified physician. Novartis is not a formal part of this MOU. On the issue of informed consent, there is a consent form signed by each patient prior to being approved in GIPAP.

Note 4. Patients with a GIST diagnosis have been excluded from the analysis.

Note 5. Both "active" and "closed" cases. Closed cases are subject to review, as patients who may be "closed" on one occasion may be re-instated subsequently.

Note 6. According to the IRIS clinical trial, estimated rates of freedom from progression to accelerated phase and blast phase or overall survival at six years were 61% and 76% respectively.
